# Analytical method for reconstructing the stress on a spherical particle from its surface deformation

**DOI:** 10.1016/j.bpj.2024.01.017

**Published:** 2024-01-22

**Authors:** Lea Johanna Krüger, Michael te Vrugt, Stephan Bröker, Bernhard Wallmeyer, Timo Betz, Raphael Wittkowski

**Affiliations:** 1 Institute of Theoretical Physics, Center for Soft Nanoscience, University of Münster, Münster, Germany; 2 DAMTP, Centre for Mathematical Sciences, University of Cambridge, Cambridge, UK; 3 Centre for Molecular Biology of Inflammation, Institute of Cell Biology, University of Münster, Münster, Germany; 4 Third Institute of Physics - Biophysics, University of Göttingen, Göttingen, Germany

## Abstract

The mechanical forces that cells experience from the tissue surrounding them are crucial for their behavior and development. Experimental studies of such mechanical forces require a method for measuring them. A widely used approach in this context is bead deformation analysis, where spherical particles are embedded into the tissue. The deformation of the particles then allows to reconstruct the mechanical stress acting on them. Existing approaches for this reconstruction are either very time-consuming or not sufficiently general. In this article, we present an analytical approach to this problem based on an expansion in solid spherical harmonics that allows us to find the complete stress tensor describing the stress acting on the tissue. Our approach is based on the linear theory of elasticity and uses an ansatz specifically designed for deformed spherical bodies. We clarify the conditions under which this ansatz can be used, making our results useful also for other contexts in which this ansatz is employed. Our method can be applied to arbitrary radial particle deformations and requires a very low computational effort. The usefulness of the method is demonstrated by an application to experimental data.

## Significance

Measurements of mechanical forces acting on cells in a tissue are important for understanding the physical behavior of biological systems, but they are also quite challenging. A common strategy is to place a spherical bead inside the tissue and to then reconstruct the mechanical stress from the bead deformation that this stress causes. Here, we introduce a novel analytical method by which this reconstruction can be achieved. This method is significantly faster than numerical approaches and significantly more general than existing analytical techniques, such that it can be expected to find a broad range of applications in mechanobiology.

## Introduction

**Figure 1:**
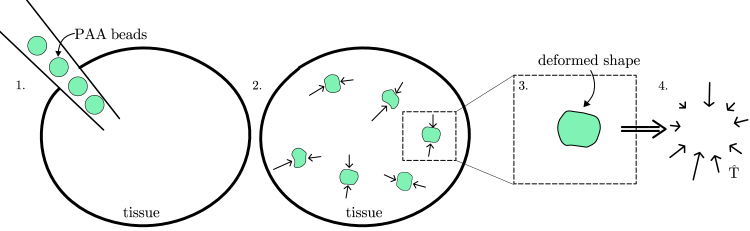
Schematic visualization of the method of bead deformation analysis. Step 1: the PAA beads are inserted into the tissue. Step 2: the beads are deformed due to stresses acting in the tissue. Step 3: using confocal microscopy, the shape of the deformed beads is obtained. Step 4: the stress tensor $\hat{T}$ is reconstructed from the deformed shape. To see this figure in color, go online.

A proper description of cell development is essential for many areas of research, including tumor research (1,2,3), embryogenesis (4), and the study of tissue growth (5,6). The development of cells is influenced by the extracellular matrix (ECM), which is a three-dimensional network of macromolecules surrounding the cells. Biochemical and mechanical properties (7,8) of the ECM alter the shape and activity of the cells (9,10,11,12,13), while the macromolecules making up the ECM are produced by the cells themselves. This leads to a feedback loop between the ECM and the cells that usually results in homeostasis. Due to the importance of the ECM, a throughout analysis of its properties is essential for understanding cell development, where homeostasis is disturbed. There has been a significant amount of work on the biochemical properties of the ECM in the past (see (10,14,15) for reviews). More recently, experimental advances made it possible to study also the mechanical properties (7,16,17) and its influences (6,18,19,20,21,22) on cell development. Measuring the forces inside the ECM is very difficult for a variety of reasons. First, the order of magnitude of the forces inside the tissue is usually in the range of pN to nN (23). Therefore, measurements have to be sensitive to very small stress differences. Second, the measurements should be performed in vivo, without disturbing the complex mechanisms inside the tissue. An innovative method developed by Campàs et al. (24) achieves this by exploiting the fact that stress acting on a soft particle results in deformation. Oil droplets are inserted into the tissue, where their deformed shape is measured. Since the mechanical properties of the oil droplets are known, the method allows to reconstruct the stress acting inside the tissue. A remaining challenge is the incompressibility of oil droplets preventing a direct measurement of compressive stresses. The idea was developed further by other researchers (16,17,23,25,26) who designed new beads whose mechanical properties are optimized for measuring the stress inside the tissue. These beads typically consist of some type of hydrogels, such as polyacrylamide (PAA). One of the main differences between these beads and oil droplets is that the beads are compressible. The general procedure is the same for all approaches where PAA beads are used (16,17,23,25,26). One places the PAA beads inside the tissue, where they are deformed due to the stresses acting on them. Then, the deformed shape is measured, usually via confocal microscopy (23). The last step is the reconstruction of the stress from the shape of the deformed bead. This last step is the main focus of this article. The individual steps are illustrated in Fig. 1. It should be noted that the different mechanical properties of bead and cells could change the stress response inside the ECM. Such a possible mismatch can be avoided by using beads whose stiffness is similar to that of a cell. Given that beads can be created with variable stiffness, such effects can be systematically checked if required. Using the theory of elasticity, the problem of reconstructing the stress from the shape deformation can be formulated as a differential equation that can be solved with the displacement of the surface as a boundary condition. Numerical approaches to this problem are computationally very expensive, and an analytical solution method applicable to arbitrarily deformed spheres does not exist. In this article, we present an improved analytical solution for the differential equation that allows to compute the stress. To achieve this, an ansatz derived by Love (27) (appearing also in (28,29)) is used. The way this ansatz is constructed leads to several subtleties concerning the harmonicity of the individual terms appearing in the ansatz. These subtleties have not been addressed in existing analytical approaches. In this article, we develop a method that allows using the ansatz for arbitrary radial bead deformations. We demonstrate the applicability of this method by an analysis of experimental data. This article is structured as follows.

In theoretical background, a short introduction to the linear theory of elasticity, including the differential equation central to this article, is provided. Current state of research contains a summary of the current state of research on the stress reconstruction problem. In ansatz and outline of the approach, the ansatz by Love is presented. In addition, we explain our general approach for solving the differential equation using the ansatz. In radial deformation and the boundary condition, the derivation of the stress tensor using the ansatz is shown. This result is, in analyzing experimental data, used to analyze example experimental data. Finally, we present our conclusions.

### Theoretical background

The reconstruction of the stress acting on a bead is possible using the theory of elasticity. In this section, the problem is formulated in the language of this theory by deriving a differential equation for the displacement vector u. Apart from the displacement vector itself, the most important quantities are the stress tensor $\hat{T}$ and the strain tensor $\hat{\epsilon }$. For investigating these quantities, we use the linear theory of elasticity, which assumes that the change of displacement $u_{i}$ in any direction is small compared with the size of the considered objects:

(1) $$\left|\frac{\partial u_{i}}{\partial x_{j}}\right|\ll 1\text{.}$$

To derive the differential equation for the displacement vector u, only a handful of fundamental relations of linear elasticity theory are needed (29,30,31). One is Hooke’s law (30)

(2) $$\hat{T}=2G\frac{\nu }{1-2\nu }\text{tr}\left(\hat{\epsilon }\right)+2G\hat{\epsilon }\text{,}$$

which linearly connects the stress tensor $\hat{T}$ and the strain tensor $\hat{\epsilon }$. Here, *G* denotes the shear modulus and ν Poisson’s ratio, both of which characterize the material properties of the deformed body. Moreover, $\text{tr}\left(\hat{\epsilon }\right)$ describes the trace of strain $\hat{\epsilon }$. Equation 2 assumes elastic, isotropic, and thermodynamically reversible deformations. Now, the strain tensor $\hat{\epsilon }$ is replaced by its definition in terms of the displacement vector u. This definition is, in the linear theory of elasticity, given by

(3) $$\hat{\epsilon }=\frac{1}{2}\left(\nabla u+{\left(\nabla u\right)}^{T}\right)=\mathrm{Def}\left(u\right)\text{,}$$

where Def(u) is the deformation of the displacement vector field. For the next step, we use the fact that the shape of the beads inside the tissue remains stable, which suggests that static equilibrium is reached. In equilibrium, the divergence of the stress tensor $\nabla \cdot \hat{T}$ equals the external forces per unit volume ρK, where ρ is the mass density of the elastic body and K the force acting on a unit mass. We assume that the external forces are negligible compared with the stress arising from the ECM. Consequently, the divergence of Eq. 2 is equal to zero. This gives the differential equation

(4) $$\frac{1}{1-2\nu }\nabla \left(\nabla \cdot u\right)+\nabla^{2}u=0\text{,}$$

from which the displacement vector field u can be calculated. In experiments, one obtains the shape of the surface of the deformed bead. If the displacement vector on the surface of the bead can be obtained from this information, it can be used as a boundary condition for solving Eq. 4, which in turn allows to obtain the entire displacement field. Inserting the result into Eqs. 2 and 3 then gives the complete stress tensor $\hat{T}$ and strain tensor $\hat{\epsilon }$ under the assumption of small and fully radial deformations.

### Current state of research

There exist a variety of methods for reconstructing the stress from the shape of the deformed bead. These face three main challenges, namely the *computational effort* needed to find the solution of Eq. 4, the *generality of shapes* the method can be applied to, and the *connection of displacement and shape*. The issue of computational effort arises primarily for numerical approaches. Solving Eq. 4 or a similar differential equation using the finite element method (FEM) allows to find a solution for arbitrary shapes of the deformed bead (25,26). The downside of the FEM and of numerical approaches in general is the computational effort. Currently, this method takes several days per bead to obtain the result (32). Taking into account that understanding the stress inside the ECM requires analyzing a large number of beads, a quicker method to find the stress is required. Alternatively, analytical approaches can be used, which are usually a lot quicker than the FEM. The constraint of many existing analytical approaches is that they make highly simplifying assumptions regarding the shape of the deformed bead. As a result, these methods do not apply to general deformations, but only to those occurring in a specific kind of experimental setup. Examples are the method by Dolega et al. (16), which only applies to hydrostatically compressed beads, and the one by Lee et al. (17)), which exclusively describes deformations along one axis. The last issue concerning the connection of displacement u and shape arises when the boundary condition for Eq. 4 is formulated. The initial state is considered to be a sphere of initial radius $r_{0}$. Equation 4 is a differential equation for the displacement u. The boundary condition should therefore contain information about u, while the only quantity known from measurement is the shape of the surface of the deformed bead. The connection between the shape of the surface and the displacement of initial the surface $u{|}_{r_{0}}$ is, however, not unique. In fact, there are infinitely many possibilities for the displacement field on the boundary $u{|}_{r_{0}}$ that result in the same shape. Often, this problem is solved by assuming that the displacement of the surface is exclusively directed into the radial direction (16,25,23). Another, more general, possibility was presented by Vorselen et al. (32), who introduced a cost function that minimizes the elastic energy to find the optimal displacement numerically.

### Ansatz and outline of the approach

Here, we introduce an analytical approach where the computational effort is low even though it applies to complicated radial deformations. This is achieved using an ansatz that solves Eq. 4 and allows a decomposition of the deformed shape into solid spherical harmonic (SHH) functions. The main ideas follow (18,23), where the same ansatz is applied. The ansatz will be analyzed further so that its implications can be understood in more depth, which allows us to use it for a generalized shape of the deformed bead. The connection of shape and displacement $u{|}_{r_{0}}$ will be assumed to be radial for now. A typical procedure for solving a differential equation of the form (4) is to use an ansatz that consists exclusively of harmonic functions (27,28,33,34). A harmonic is a function *f* that solves Laplace’s equation

(5) $$\nabla^{2}f=0\text{.}$$

Harmonic functions are useful because their behavior is well understood. In particular, it is known that harmonic functions can be expanded into SSHs, which are three-dimensional harmonic functions. There are two distinct types of SSHs, regular ($R_{n}^{m}$) and irregular ($I_{n}^{m}$) ones. They are defined as

(6) $$R_{n}^{m}\left(r,\theta ,\phi \right)=r^{n}Y_{nm}\left(\theta ,\phi \right)\text{,}$$

(7) $$I_{n}^{m}\left(r,\theta ,\phi \right)=\frac{1}{r^{n+1}}Y_{nm}\left(\theta ,\phi \right)\text{,}$$

where $Y_{nm}$ are spherical harmonic functions (see Appendix B for the definition used in this article). The difference between regular and irregular SSHs is that regular SSHs diverge for large *r*, whereas irregular SSHs diverge at the origin. Equation 4 can be solved using a general expansion of an ansatz for u, consisting of harmonic functions, into SSHs. The coefficients of the expansion have to be deduced from the boundary conditions of the problem under consideration. A procedure of this form is useful for the bead deformation analysis because the shape of the deformed bead is usually expanded into spherical harmonics as well. The maximal order of this expansion determines the precision of the measurement. One ansatz for the displacement u that solves Eq. 4 and consists of harmonic functions only was suggested by Love (27) and Trefftz (28). The ansatz is particularly useful for studying the deformation of spherical bodies and is given by

(8) $$u\left(r,\theta ,\phi \right)=U\left(r,\theta ,\phi \right)+\left(r^{2}-r_{0}^{2}\right)\nabla \psi \left(r,\theta ,\phi \right)\text{,}$$

where ψ and all components of the vector function U are harmonic functions. Moreover, $r_{0}$ is the radius of the initial spherical body and *r* is the radial coordinate, where the origin lies in the center of the initial sphere. Equation 8 shows that the function u coincides with U on the surface of the initial sphere (i.e., for $r=r_{0}$). From Eq. 8, a connection of the scalar function ψ and the vector function U can be deduced. This relation is based on an expansion of both functions ψ and U into SSHs and connects $\Psi_{n-1}$ and $U_{n}$, where *n* is the order of the corresponding SSH:

(9) $$\psi_{n-1}=\frac{\nabla \cdot U_{n}}{-2\left(\left(2-3n\right)-2\nu \left(1-2n\right)\right)}\text{.}$$

The derivation of Eqs. 8 and 9 is shown in Appendix A. This derivation shows that Eq. 9 is only valid if U is expanded to $U_{n}$ in the Cartesian basis. If all Cartesian components of $U_{n}$ are SSHs of order *n*, the same cannot be true for the spherical components of $U_{n}$. In fact, Eq. 9 can only work in the way stated above if $U_{n}$ is an SSH of order *n* in all of its Cartesian components. Having understood the conditions for the applicability of the ansatz given by Eq. 8, we now expand the complete displacement function u as given in Eq. 8 into SSHs to obtain the general solution of Eq. 4, where the coefficients of the expansion still need to be evaluated. For the expansion of the scalar function ψ and vector function U, regular SSHs are chosen inside the initial sphere and irregular SSHs outside it. Therefore, the deformation vector u does not diverge for large *r* and is not singular at the origin. In addition, the expansion is ascertained to be continuous by the use of appropriate prefactors (23,29):

(10) $$\psi =\left\{ \begin{array}{cc} \sum_{n=0}^{\infty }\sum_{m=-n}^{n}\frac{r^{n}}{r_{0}^{n}}a_{nm}Y_{nm} & \text{for}\;r<r_{0},\\ \sum_{n=0}^{\infty }\sum_{m=-n}^{n}\frac{r_{0}^{n+1}}{r^{n+1}}a_{nm}Y_{nm} & \text{for}\;r>r_{0}, \end{array}\right.$$

(11) $$U=\left\{ \begin{array}{cc} \sum_{n=0}^{\infty }\sum_{m=-n}^{n}\frac{r^{n}}{r_{0}^{n}}K_{nm}Y_{nm} & \text{for}\;r<r_{0},\\ \sum_{n=0}^{\infty }\sum_{m=-n}^{n}\frac{r_{0}^{n+1}}{r^{n+1}}K_{nm}Y_{nm} & \text{for}\;r>r_{0}. \end{array}\right.$$

What is missing for the full solution of Eq. 4 are the scalar coefficients $a_{nm}$ and the vectorial coefficients $K_{nm}$. If these coefficients are known, the complete displacement field u(r,θ,ϕ) is determined. Inserting Eq. 8 into Eq. 3 and the result into Hooke’s law gives the complete stress tensor $\hat{T}$ in terms of ψ and U:

(12) $$\hat{T}=2G(\frac{\nu }{1-2\nu }\left(\nabla \cdot U+2r\cdot \nabla \psi \right)\hat{E}+\mathrm{Def}\left(U\right)+r⊗\nabla \psi +{\left(r⊗\nabla \psi \right)}^{T}+\left(r^{2}-r_{0}^{2}\right)\nabla ⊗\nabla \psi )\text{.}$$

Here, r is the positional vector, $\hat{E}$ is the three dimensional unit tensor, and ⊗ is the outer product ${\left(u⊗v\right)}_{ij}=u_{i}v_{j}$.

### Radial deformation and the boundary condition

To obtain the coefficients $a_{nm}$ and $K_{nm}$ that fully determine the stress tensor $\hat{T}$, we require the boundary condition for the displacement vector $u{|}_{r_{0}}$. The experimental data only provide the shape of the deformed beads. To construct a boundary condition for the displacement vector from this information, we assume that the displacement is only radial. For the experimental setup considered here, this assumption can be assumed to be reasonably accurate for small deformations. Since the beads are based on a fully inert PAA hydrogel, no attachment between the cells and the beads is possible, because cells cannot bind PAA. Therefore, all forces acting on the bead should be acting normal to its surface. Note, however, that more complicated displacement fields are also possible if there are shear stresses. For such deformations, our method is not applicable in its present form. If the radius of the deformed bead at angles *θ* and *ϕ* is given by s(θ,ϕ), this deformed shape can be expanded into spherical harmonics (see Appendix B for the definition used in this article) in the form

(13) $$s\left(\theta ,\phi \right)=\sum_{n=0}^{N}\sum_{m=-n}^{n}s_{nm}Y_{nm}\text{,}$$

where the expansion coefficients $s_{nm}$ are given by

(14) $$s_{nm}=\int_{\Omega }s\left(\theta ,\phi \right)Y_{nm}^{*}\left(\theta ,\phi \right)d\Omega \text{.}$$

In practice, the expansion needs to be truncated at some order *N*. Therefore, shapes that exhibit sharp corners cannot be approximated by Eq. 13 with sufficient accuracy. Since we are interested here primarily in deformations occurring for a bead placed inside biological tissue, it is a reasonable assumption that the deformations are sufficiently smooth to allow for a truncation of this expansion. In other contexts, where sharp corners appear, such a truncation would not be accurate. We then assume the surface deformation to be

(15) $$u\left(r_{0},\theta ,\phi \right)=\left(r_{0}-s\left(\theta ,\phi \right)\right){\hat{e}}_{r}=\sum_{n=0}^{N}\sum_{m=-n}^{n}d_{n,m}Y_{nm}{\hat{e}}_{r}\text{,}$$

where $r_{0}$ is the radius of the undeformed bead, ${\hat{e}}_{r}$ is the unit vector in radial direction and

(16) $$d_{n,m}=\left\{ \begin{array}{ll} \sqrt{4\pi }r_{0}-s_{00} & \text{for}\;n=m=0,\\ -s_{nm} & \text{else} \end{array}\right.$$

are the coefficients resulting from the radial difference of the initial sphere and the shape of the deformed bead s(θ,ϕ). At this point, the importance of the prerequisites of the ansatz come into play. Because of the way the ansatz in Eq. 8 is constructed, the displacement of the surface completely defines the coefficients of the harmonic vector function U. To use Eq. 9 to determine the coefficients $a_{nm}$ that define the function ψ, we require the harmonic expansion of U in terms of Cartesian coordinates. In Cartesian coordinates, the radial unit vector ${\hat{e}}_{r}$ consists of spherical harmonic functions of order 1:

(17) $${\hat{e}}_{r}=\left(\begin{array}{c} Y_{1-1}-Y_{11}\\ i\left(Y_{1-1}+Y_{11}\right)\\ \sqrt{2}Y_{10}. \end{array}\right)\text{.}$$

To obtain the required expansion of $u{|}_{r_{0}}$, we need to express the product of spherical harmonics $Y_{nm}$ occurring in the Cartesian components in Eq. 15 in harmonic functions. A useful relation for this purpose is (35)

(18) $$\begin{array}{l} Y_{l_{1}}^{m_{1}}Y_{l_{2}}^{m_{2}}=\sqrt{4\pi }\sum_{L=\left|l_{1}-l_{2}\right|}^{\left|l_{1}+l_{2}\right|}C_{l_{1},m_{1},l_{2},m_{2}}^{L,M}C_{l_{1},0,l_{2},0}^{L,0}\\ \sqrt{\frac{\left(2l_{1}+1\right)\left(2l_{2}+1\right)}{2L+1}}Y_{L}^{M}\text{,} \end{array}$$

where $C_{l_{1},m_{1},l_{2},m_{2}}$ are Clebsch-Gordan coefficients and $M=m_{1}+m_{2}$. Using Eq. 18, the boundary condition $u{|}_{r_{0}}$ is expressed in such a way that the *n*th term of the expansion contains the spherical harmonic of degree *n* in every Cartesian component. One can then directly express the coefficients of this expansion with the vectorial coefficients $K_{nm}$ occurring in Eq. 11 in terms of the known coefficients $d_{n,m}$ and the appropriate Clebsch-Gordan coefficients (Appendix C). Without transforming the boundary condition given by Eq. 15 into Cartesian coordinates, one cannot make proper use of the ansatz given by Eq. 8. Having determined the coefficients $K_{nm}$, we can construct the harmonic vector function U using Eq. 11. To use Eq. 9 and to obtain the coefficients $a_{nm}$ that describe the harmonic scalar function ψ, one needs to evaluate the divergence of U. A general expression for the divergence of U can only be obtained if the individual derivatives of the components U are expressed as an expansion in SSHs. The derivatives of spherical harmonic functions with respect to Cartesian coordinates are given in Appendix D. These allow to express the divergence of U in terms of the coefficients $K_{nm}$, which is done in Eq. D4 in Appendix D. Applying the method of equating the coefficients to Eqs. 9 and D4 yields

(19) $$a_{n-1m}=\frac{c_{nm}}{2\left(2\nu \left(2n-1\right)-3n\right)}\text{,}$$

where we introduced the coefficients $c_{nm}$ to keep the expression in Eq. 19 short. The derivation of Eq. 19 and the definition of the coefficients $c_{nm}$ in terms of $K_{nm}$ is given in Appendix D. Thereby, we have determined the functions ψ and U as given in Eqs. 10 and 11 from the boundary condition given by Eq. 15. We now insert the results for ψ and U into Eq. 12 to obtain the full stress tensor:

(20) $$\hat{T}\left(r,\theta ,\phi \right)=\sum_{n=0}^{N}\sum_{m=-n}^{n}\frac{2G}{r_{0}}\{\frac{\nu }{1-2\nu }a_{nm}\left(2r_{0}\left(1-3n+2\nu \left(2n+1\right)\right)r^{n}Y_{nm}+2r\cdot \nabla \left(r^{n}Y_{nm}\right)\right)+\left(\left(K_{nm}\cdot e_{r}\right)e_{r}+\left(K_{nm}\cdot e_{\theta }\right)e_{\theta }+\left(K_{nm}\cdot e_{\phi }\right)e_{\phi }\right)⊗\nabla \left(r^{n}Y_{nm}\right)+\nabla \left(r^{n}Y_{nm}\right)⊗\left(\left(K_{nm}\cdot e_{r}\right)e_{r}+\left(K_{nm}\cdot e_{\theta }\right)e_{\theta }+\left(K_{nm}\cdot e_{\phi }\right)e_{\phi }\right)+\left(r^{2}+r_{0}^{2}\right)a_{nm}\nabla ⊗\nabla \left(r^{n}Y_{nm}\right)\}\text{.}$$

where $e_{\theta }$ is the azimuthal and $e_{\phi }$ the polar unit vector. Even though the expression in Eq. 20 is rather long, the evaluation for an arbitrarily chosen bead is simple once the tensor has been evaluated up to the desired order. The overall form of the stress tensor remains the same for every bead and depends on the coefficients $a_{nm}$ and $K_{nm}$ explicitly. Therefore, the effort of analyzing multiple beads is greatly reduced compared with numerical methods. $a_{nm}$ is obtained by inserting the coefficients $d_{n,m}$ (Eq. 16), which follow from the deformed shape into Eq. D5 and the resulting coefficients $c_{nm}$ into Eq. 9. The coefficients $K_{nm}$ follow from inserting $d_{n,m}$ into (C1), (C2), (C3). Meanwhile, the result allows—in contrast to previously developed analytical approaches—to examine beads of arbitrary radial deformation. For more general (nonradial) deformations, the result shown in Eq. 20 is subject to limitations. We now analyze the properties of the stress tensor shown in Eq. 20. To better understand the result, we consider some basic limiting cases. If the deformed bead remains completely spherical, the resulting stress tensor is proportional to the unit tensor $\hat{E}$. The magnitude of the stress is then fully determined by Poisson’s ratio ν, the shear modulus *G*, and the difference between the initial radius $r_{0}$ and the radius of the deformed sphere $r_{1}$:

(21) $${\hat{T}}_{\text{sphere}}=8G\pi \frac{\left(r_{1}-r_{0}\right)\left(1+\nu \right)}{r_{0}\left(1-2\nu \right)}\hat{E}\text{.}$$

A uniformly deformed bead therefore corresponds to an isotropic stress tensor. Another case that is relevant for many experimental setups is a uniaxial deformation, where the bead is deformed only in one particular direction. Here, we consider as an example a shape function of the deformed bead $s_{\text{uniaxial}}\left(\phi ,\theta \right)$ that is given as a sum of the initial sphere (described by the zeroth-order spherical harmonic $Y_{00}$, which is a constant) and an additional term containing the spherical harmonic function of order 2 with m=0:

(22) $$s_{\text{uniaxial}}=\sqrt{4\pi }r_{0}Y_{00}+d_{2,0}Y_{20}=s_{\text{initial}}+d_{2,0}Y_{20}\text{.}$$

**Figure 2:**
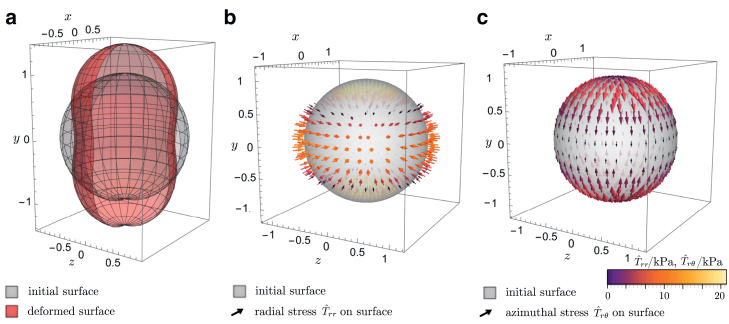
The case of uniaxial deformation. (*a*) Shape of the initial sphere of unit radius compared with the deformed shape of the bead as given in Eq. 22 with $d_{2,0}=0.2$. (*b*) Radial stress and (*c*) azimuthal stress on the surface of the initial sphere, with the arrows indicating the direction and magnitude of the stress. To see this figure in color, go online.

A visualization of the initial sphere $s_{\text{initial}}=\sqrt{4\pi }r_{0}Y_{00}$ and the deformed sphere is given in Fig. 2. Using Eq. 22 to determine the coefficients $a_{nm}$ and $K_{nm}$ by Eqs. 19 and (C1), (C2), (C3) and inserting these into Eq. 20, we obtain a stress tensor that consists of a radial term and an azimuthal term. The radial stress is proportional to the radial displacement of the initial sphere compared with the deformed sphere, similar to the case of spherical deformation. The azimuthal stress, on the other hand, depends on the change of the difference of radii along the azimuthal direction. Both components of the stress tensor are plotted on the surface of the initial sphere in Fig. 2. For displacements that are not uniaxial, an additional polar term appears which represents the stress acting on the sphere in polar direction. Similar to the azimuthal stress, the polar stress depends on the change of the difference of radii along the azimuthal direction.

### Analyzing experimental data

So far, we have discussed very simple shapes where only a small number of spherical harmonics is required to describe the shape of the deformed bead. The stress tensor derived here (Eq. 20) is, however, applicable to arbitrary radial bead deformations. To test this result for more complicated cases, experimental data are obtained and analyzed.

**Figure 3:**
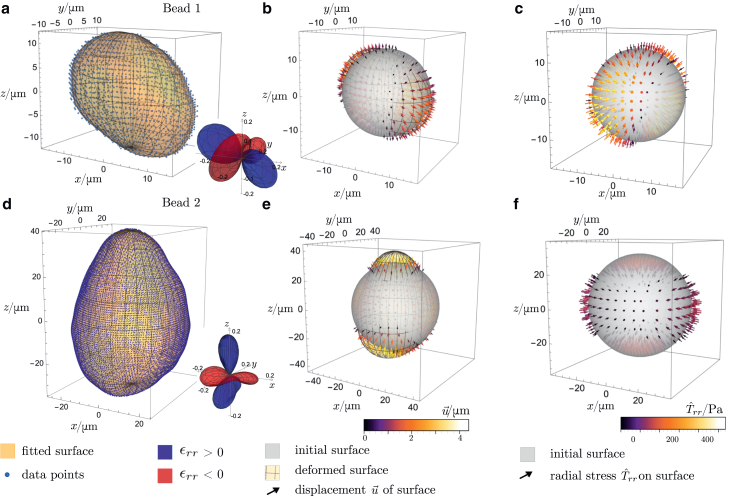
Analysis of experimental data for the deformation of two PAA beads. (*a*) Data points and fitted reconstructed surface of bead 1. The order of fitting is $n_{\max}=13$. We depict the magnitude of the normal strain $\left|\epsilon_{rr}\right|$ in the inset, where positive strain is given in blue and negative in red. (*b*) Initial and deformed surfaces of bead 1. The arrows represent the displacement u. (*c*) Radial stress on the surface of the undeformed bead 1. (*d*)–(*f*) Like (*a*)–(*c*), but for bead 2. The order of fitting is $n_{\max}=26$ now. To see this figure in color, go online.

In the experiments, beads consisting of PAA were marked by fluorescent pigments and then embedded into the tissue, where the shape of their surface was measured using a light-sheet microscope. A detailed description of the experimental setup can be found in section 3.2 of (23). The material properties (Poisson’s ratio ν and shear modulus *G*) of the beads are measured to be ν=0.491±0.005 and G=720±270 Pa (23,36). For this experimental setup, the original size of the beads varies and cannot be determined after the beads are inserted into the tissue. We assume the beads to be incompressible and perfectly spherical to reconstruct the initial shape of the spheres from the available data. As the Poisson’s ratio ν of the beads is close to, but not exactly 0.5, it can be seen that the beads are not entirely incompressible. The assumption that the beads are fully incompressible will therefore only be used to find the initial shape and will not be exploited for determining the stress (since our method is applicable also in the compressible case). In general, the analytical method developed here is applicable to compressible beads and no assumption of incompressibility is needed if the initial shape and size of the beads are known. The first step of analyzing the data is to numerically fit a spherical harmonic expansion to the data points for the bead’s surface. To determine a sufficiently high upper order for fitting while avoiding overfitting, the data points are compared with the surface generated by the fit. The order of the fit is increased until the order $2n_{\max}$, where the fit exactly coincides with the data points. The more data points there are, the higher the order of the fit needs to be to achieve this. At this order, the fitted surface in between the data points is not as smooth as it should be, implying that the data are overfitted. To avoid overfitting, we truncate the expansion at order $n_{\max}$. This constitutes a reasonable compromise since at order $n_{\max}$ the data points are fitted with reasonable accuracy, but the curve between them is still smooth (as can be seen in Fig. 3). The number of data points differs for the individual beads, implying that $n_{\max}$ differs as well. In Fig. 3, *a*–*d*, we show data points and fits for two of the analyzed beads. The maximal order of fitting is $n_{\max}=13$ for the first bead (Fig. 3 *a*) and $n_{\max}=26$ for the second bead (Fig. 3 *d*). As can be seen (Fig. 3, *a* and *d*) both fitted surfaces resemble the data points smoothly. As a next step, the original size of the beads is determined by evaluating the volume enclosed by the fitted surface. The original bead is now described by a sphere of the same volume. Thereby, a mathematical description of the shape of the original and the deformed particle is known. Using Eq. 15, we evaluate the displacement $u\left(r,\theta ,\phi \right){|}_{r_{0}}$ of the surface. The coefficients $a_{nm}$ and $K_{nm}$ are determined using the procedure explained in radial deformation and the boundary condition. Then, the resulting coefficients are inserted into the expansion of the stress tensor given by Eq. 12. As a result, we obtain the complete stress tensor for the beads. To visualize some example results, the radial stress ${\hat{T}}_{rr}$ on the surface of the original beads is plotted for the two beads in Fig. 3, *c*–*f*. In addition, the radial displacement of the surface (Eq. 15) is plotted in comparison with the radial stress (Fig. 3, *b*–*e*). Further components of the stress tensor could have been calculated as well. Here, only two components were chosen to visualize the results. The stress is pointing inwards when the bead is compressed and outwards when it is stretched. Therefore, the method introduced in this work allows to reconstruct the stress exerted on radially deformed spherical particles even if the shape of the deformed particle is complicated, as it typically is in experimental contexts. Since our method is based on the linear theory of elasticity, the assumption of small displacements compared with the object size as given in Eq. 1 needs to be fulfilled by the analyzed beads. For the beads in the present experiment, the maximal normal strain is $\epsilon_{rr}\approx$ 0.2 as can be seen in the depicted inset (Fig. 3, *a* and *d*). For these values, the linear theory of elasticity should be a good approximation.

## Conclusions

In this article, a general analytical approach that allows to reconstruct the complete stress tensor for any radially deformed spherical bead from the deformation of its surface was derived in the framework of linear elasticity. Compared with numerical calculations of the stress tensor, the analytical evaluation is significantly faster. Since the result is given in terms of an expansion into SSHs, the overall form of the result to a particular order is the same for all beads, only differing by the coefficients of expansion. The evaluation of those coefficients, resulting from expansion coefficients of the deformed bead, takes only a few seconds or less on a computer. Moreover, the effort does not increase significantly if more than one bead has to be analyzed, whereas a numerical solution for every single bead can take up to several days (32). As the method is based on the linear theory of elasticity, the beads should be chosen in such a way that their deformations are sufficiently small to ensure the applicability of this theory, but sufficiently large to allow for precise measurements. In this work, the displacement field was assumed to be fully radial to overcome the problem that the displacement field is not uniquely determined by the experimental data. For PAA beads, which do not bind to the cells, this is the most natural approach for small deformations. Our analytical method could also be extended to more general, nonradial, deformations. An interesting first step in this context would be an analysis of the relevance of shear stresses for small deformations, for example, by using an energy minimization approach. Moreover, our approach could be extended to nonlinear elasticity theory.

## Data and code availability

The experimental data analyzed in this study (cf. Fig. 3) and the spherical harmonics expansion coefficients used for fitting these data are available online (37).

## Author contributions

L.J.K. performed the derivation. B.W. measured the experimental data. L.J.K. and S.B. analyzed the data and prepared the first version of the figures. L.J.K. and M.t.V. wrote the first version of the text. T.B. and R.W. conceived the project and revised the text. M.t.V., T.B., and R.W. revised the figures and supervised the work.

## Appendix A: Derivation of THE ANSATZ

In this appendix, we discuss the derivation of Eq. 8. The discussion follows Love (27), but uses a modern notation and focuses on aspects relevant for the present work. To solve Eq. 4, a harmonic representation for the complete displacement $u\left(r_{,}\theta ,\phi \right)$ is derived.

By evaluating the divergence of Eq. 4 and using the fact that the Laplacian $\nabla^{2}=\nabla \cdot \nabla$ and the divergence commute, it can be shown that the divergence of u is a harmonic function:

(A1) $$0=\frac{2-2\nu }{1-2\nu }\nabla^{2}\left(\nabla \cdot u\right)\text{.}$$

Harmonic functions can be expanded into SSHs. Therefore, ∇·u is known to be of the form

(A2) $$\nabla \cdot u=\sum_{n=0}^{\infty }\Theta_{n}\text{,}$$

where $\Theta_{n}$ are SSHs of degree *n*. Equation 4 can then be written in terms of the Laplacian of the displacement u and the expansion of ∇·u:

(A3) $$\nabla^{2}u=\frac{1}{2\nu -1}\sum_{n=0}^{\infty }\nabla \Theta_{n}\text{.}$$

If Eq. 4 is written in this form, one can see that it can be solved by finding a particular solution $u_{p}$ for Eq. A3 and a complementary solution for $\nabla^{2}u_{c}=0$. Obviously, the complementary solution $u_{c}$ is also a harmonic function. A particular solution $u_{p}$ to Eq. A3 is obtained by evaluating the Laplacian of $r^{2}\nabla \Theta_{n}$. It is used here that the Laplacian of a harmonic function vanishes:

(A4) $$\nabla^{2}\left(r^{2}\nabla \Theta_{n}\right)=6\nabla \Theta_{n}+\left(4r\cdot \nabla \right)\left(\nabla \Theta_{n}\right)=6\nabla \Theta_{n}+4\left(n-1\right)\nabla \Theta_{n}=\left(4n+2\right)\nabla \Theta_{n}\text{.}$$

If we express the harmonic functions $\Theta_{n}$ in terms of Cartesian coordinates, every component of $\nabla \Theta_{n}$ is also a homogeneous function of degree n−1 (27). This is used in the second step of Eq. A4, where Euler’s theorem on homogeneous functions is applied even though it does not hold for spherical components of SSHs. Therefore, from this point on the derivation does not work for expansions of $\nabla \Theta_{n}$ in curvilinear coordinates. Using Eq. A4, a particular solution $u_{p}$ to Eq. A3 can be constructed:

(A5) $$u_{p}=\frac{1}{2\nu -1}r^{2}\sum_{n=0}^{\infty }\frac{1}{2\left(2n+1\right)}\nabla \Theta_{n}\text{.}$$

The complete solution for u has to satisfy the condition imposed by choosing $\nabla \cdot u=\sum_{n}\Theta_{n}$. Using $u=u_{p}+u_{c}$, this gives

(A6) $$\nabla \cdot \left(u_{p}+u_{c}\right)=\sum_{n=0}^{\infty }\Theta_{n}\text{.}$$

The particular solution (Eq. A5), which is written in terms of $\Theta_{n}$, is inserted into Eq. A3. As regular spherical harmonics of degree *n* are also homogeneous of degree *n*, Euler’s theorem on homogeneous functions can be used:

(A7) $$\nabla \cdot u_{c}=\sum_{n=0}^{\infty }\left(\frac{3n+1-2\nu \left(1+2n\right)}{\left(2n+1\right)\left(1-2\nu \right)}\Theta_{n}\right)\text{.}$$

As a result, the complete deformation vector u can be expressed in terms of the complementary solution $u_{c}$. If $u_{c,n}$ is the part of $u_{c}$ which is homogeneous of degree *n* and therefore consists of SSHs in all of its Cartesian components, the particular solution $u_{p}$ can be written in terms of these harmonic functions:

(A8) $$u_{p}=\sum_{n=0}^{\infty }r^{2}\frac{-1}{2\left[-2\nu \left(2n+1\right)+3n+1\right]}\nabla \left(\nabla \cdot u_{c,n+1}\right)\equiv \sum_{n=0}^{\infty }r^{2}M_{n+1}\nabla \left(\nabla \cdot u_{c,n+1}\right)\text{,}$$

where the coefficients $M_{n}$ are introduced as a shorthand notation:

(A9) $$M_{n}=\frac{1}{2\left[2\nu \left(2n-1\right)-3n+2\right]}\text{.}$$

In the sum (Eq. A8), the *n*th term is harmonic of degree n−1. The vectors $u_{p}$ and $u_{c}$ are added to construct the complete deformation vector u in such a way that the *n*th component is harmonic of degree *n*:

(A10) $$u=\sum_{n=0}^{\infty }\left(u_{c,n}+r^{2}M_{n+2}\nabla \left(\nabla \cdot u_{c,n+2}\right)\right)\text{.}$$

The next step is to use the boundary condition for the deformation on the surface, which is specified via an expansion into spherical surface harmonics with coefficients $A_{n}$:

(A11) $$u\left(r_{0}\right)=\sum_{n=0}^{\infty }A_{n}\text{.}$$

The surface harmonical expansion (Eq. A11) is expanded again to regular solid harmonics. On the surface $\left(r=r_{0}\right)$, the difference of the derived expression (Eq. A10) for u and the expanded shape of the surface has to vanish:

(A12) $$\sum_{n=0}^{\infty }\left(u_{c,n}+r_{0}^{2}M_{n+2}\nabla \left(\nabla \cdot u_{c,n+2}\right)-\frac{r^{n}}{r_{0}^{n}}A_{n}\right)=0\text{.}$$

The expression on the left-hand side of Eq. A12 is a regular harmonic function for $r<r_{0}$ and vanishes for $r=r_{0}$. This can only be true if the expression on the left-hand side of Eq. A12 vanishes for all *r* and not only on the surface. Thereby, an expression for the *n*th component of $u_{c,n}$ is obtained:

(A13) $$u_{c,n}=r_{0}^{2}M_{n+2}\nabla \left(\nabla \cdot u_{c,n+2}\right)+\frac{r^{n}}{r_{0}^{n}}A_{n}\text{.}$$

Equation A13 is now inserted into the complete expression for the deformation vector u given by Eq. A10. We can now compare the result

(A14) $$u_{n}=\frac{r^{n}}{r_{0}^{n}}A_{n}+\left(r^{2}-r_{0}^{2}\right)M_{n+2}\nabla \left(\nabla \cdot u_{c,n+2}\right)$$

to the ansatz shown in Eq. 8. Since $r^{n}A_{n}/r_{0}^{n}$ is a harmonic vector function that is equal to the displacement u on the surface, the first term on the right-hand side in Eq. A14 is in agreement with the ansatz in Eq. 8. The expression $\nabla \cdot u_{c,n+2}$ is a harmonic scalar function of degree n+1 if $u_{c,n+2}$ is harmonic of degree n+2 in its Cartesian components. Consequently, the second term on the right-hand side of Eq. A14 can be expressed as the gradient of a harmonic scalar function multiplied by $r^{2}-r_{0}^{2}$, which exactly corresponds to the ansatz Eq. 8.

Next, we derive Eq. 9. To achieve this, we first obtain a connection between the functions $u_{c,n}$ and $r^{n}A_{n}/r_{0}^{n}$. These functions can then be expressed with ψ and U. Taking the divergence of Eq. A13 and rearranging the result gives

(A15) $$\nabla \cdot u_{c,n}=\nabla \cdot \left(\frac{r^{n}}{r_{0}^{n}}A_{n}\right)\text{.}$$

The *n*th component of the harmonic scalar function ψ is equivalent to $M_{n+1}\nabla \cdot u_{c,n+1}$. Moreover, the *n*th component of the harmonic vector function $U_{n}$ can be identified with $r^{n}A_{n}/r_{0}^{n}$. Replacing $r^{n}A_{n}/r_{0}^{n}$ and $M_{n+1}\nabla \cdot u_{c,n+1}$ by $U_{n}$ and ψ in Eq. A15, we obtain

(A16) $$\nabla \cdot U_{n}=\frac{\psi_{n-1}}{M_{n}}\text{,}$$

which can be solved for $\psi_{n-1}$. In addition, the definition of $M_{n}$ as given by Eq. A9 is inserted. This gives

(A17) $$\psi_{n-1}=\frac{\nabla \cdot U_{n}}{2\left[2\nu \left(2n-1\right)-3n+2\right]}\text{,}$$

which is equivalent to Eq. 9.

## Appendix B: Definition of spherical harmonics.

In the literature, a variety of similar but not equivalent definitions for the spherical harmonic functions are used. Therefore, we here explicitly state the convention used in this article. The spherical harmonics can be defined in terms of the associated Legendre polynomials $P_{l}^{m}$. Here, we use the normalized spherical harmonics (38)

(B1) $$Y_{l}^{m}=\left\{ \begin{array}{cc} N_{lm}P_{l}^{m}\left(\cos\;\theta \right)\;\exp\left(im\phi \right) & \text{for}\;m\geq 0,\\ {\left(-1\right)}^{m}{\left[Y_{l}^{m}\right]}^{*} & \text{for}\;m<0 \end{array}\right.$$

with the polar coordinate θ (0≤θ≤π) and the azimuthal coordinate ϕ (0≤ϕ≤2π). The factor $N_{lm}$ is given by

(B2) $$N_{lm}=\sqrt{\frac{2l+1\left(l-m\right)!}{4\pi \left(l+m\right)!}}\text{.}$$

The associated Legendre polynomials $P_{l}^{m}\left(x\right)$ are defined by the Legendre polynomials $P_{l}\left(x\right)$ as (38)

(B3) $$P_{l}^{m}\left(x\right)=\left\{ \begin{array}{cc} {\left(1-x^{2}\right)}^{\frac{m}{2}}\frac{d^{m}P_{l}\left(x\right)}{dx^{m}} & \text{for}\;m\geq 0,\\ {\left(-1\right)}^{m}\frac{\left(l-m\right)!}{\left(l+m\right)!}P_{l}^{m}\left(x\right) & \text{for}\;m<0, \end{array}\right.$$

which can be evaluated using Rodrigue’s formula (38)

(B4) $$P_{l}\left(x\right)=\frac{1}{2^{l}l!}\frac{d^{l}}{dx^{l}}{\left(x^{2}-1\right)}^{l}\text{.}$$

## Appendix C: Transforming U into Cartesian coordinates.

Here, we express the coefficients $K_{nm}$ in terms of the coefficients $d_{n,m}$ from the boundary condition and the Clebsch-Gordan coefficients, where the ${\hat{e}}_{x},{\hat{e}}_{y}$, and ${\hat{e}}_{z}$ are the unit vectors in x,y, and *z* directions, respectively:

(C1) $$K_{nm}\cdot {\hat{e}}_{x}=\sqrt{\frac{2\pi }{3}}[\left(d_{n,m+1}C_{n,m+1,1,-1}^{n,m}-d_{n,m-1}C_{n,m-1,1,1}^{n,m}\right)C_{n,0,1,0}^{n,0}\sqrt{12\pi }+\left(d_{n-1,m+1}C_{n-1,m+1,1,-1}^{n,m}-d_{n-1,m-1}C_{n-1,m-1,1,1}^{n,m}\right)C_{n-1,0,1,0}^{n,0}\sqrt{\frac{12\pi \left(2n-1\right)}{2n+1}}+\left(d_{n+1,m+1}C_{n+1,m+1,1,-1}^{n,m}-d_{n+1,m-1}C_{n+1,m-1,1,1}^{n,m}\right)C_{n+1,0,1,0}^{n,0}\sqrt{\frac{12\pi \left(2n+3\right)}{2n+1}}],$$

(C2) $$K_{nm}\cdot {\hat{e}}_{y}=i\sqrt{\frac{2\pi }{3}}[\left(d_{n,m+1}C_{n,m+1,1,-1}^{n,m}+d_{n,m-1}C_{n,m-1,1,1}^{n,m}\right)C_{n,0,1,0}^{n,0}\sqrt{12\pi }+\left(d_{n-1,m+1}C_{n-1,m+1,1,-1}^{n,m}+d_{n-1,m-1}C_{n-1,m-1,1,1}^{n,m}\right)C_{n-1,0,1,0}^{n,0}\sqrt{\frac{12\pi \left(2n-1\right)}{2n+1}}+\left(d_{n+1,m+1}C_{n+1,m+1,1,-1}^{n,m}+d_{n+1,m-1}C_{n+1,m-1,1,1}^{n,m}\right)C_{n+1,0,1,0}^{n,0}\sqrt{\frac{12\pi \left(2n+3\right)}{2n+1}}]\text{,}$$

(C3) $$K_{nm}\cdot {\hat{e}}_{z}=2\sqrt{\frac{\pi }{3}}[d_{n,m}C_{n,m,1,0}^{n,m}C_{n,0,1,0}^{n,0}\sqrt{12\pi }+d_{n-1,m}C_{n-1,m,1,0}^{n,m}C_{n-1,0,1,0}^{n,0}\sqrt{\frac{12\pi \left(2n-1\right)}{2n+1}}+d_{n+1,m}C_{n+1,m,1,0}^{n,m}C_{n+1,0,1,0}^{n,0}\sqrt{\frac{12\pi \left(2n+3\right)}{2n+1}}]\text{.}$$

## Appendix D: Derivatives of SSHs with respect to Cartesian coordinates.

The derivatives of SSHs with respect to Cartesian coordinates can be derived by using various recurrence relations for Legendre polynomials and associated Legendre polynomials. The derivatives are given by

(D1) $$\frac{\partial \left(r^{n}Y_{n}^{m}\left(\theta ,\phi \right)\right)}{\partial x}=\frac{N_{n,m}}{2}\left[\frac{Y_{n-1,m+1}}{N_{n-1,m-1}}-\left(n+m\right)\left(m-1+n\right)\frac{Y_{n-1,m-1}}{N_{n-1,m-1}}\right]\text{,}$$

(D2) $$\frac{\partial \left(r^{n}Y_{n}^{m}\left(\theta ,\phi \right)\right)}{\partial y}=\frac{N_{n,m}}{2}i\left[-\frac{Y_{n-1,m+1}}{N_{n-1,m-1}}-\left(n+m\right)\left(m-1+n\right)\frac{Y_{n-1,m-1}}{N_{n-1,m-1}}\right]\text{,}$$

(D3) $$\frac{\partial \left(r^{n}Y_{n}^{m}\left(\theta ,\phi \right)\right)}{\partial z}=\frac{N_{n,m}}{N_{n-1,m}}\left(n+m\right)Y_{n-1}^{m}\text{.}$$

Using the derivatives shown in Eqs. D1–D3, the divergence of $U_{n}$ can be easily expressed as an expansion into SSHs, where SSHs of the same order are grouped together:

(D4) $$\nabla \cdot U_{n}=\sum_{m=-n}^{n}\frac{r^{n-1}Y_{n-1,m}}{r_{0}^{n}N_{n-1,m}}\frac{1}{2}[-\left(m+n\right)\left(m+n+1\right)N_{n,m+1}\left(K_{x,n,m+1}+iK_{y,n,m+1}\right)+N_{n,m-1}\left(K_{x,n,m-1}-iK_{y,n,m-1}\right)+2N_{n,m}\left(n+m\right)K_{z,n,m}]\equiv \sum_{m=-n+1}^{n-1}\frac{r^{n-1}Y_{n-1,m}}{r_{0}^{n-1}}c_{nm}\text{,}$$

where the coefficients $c_{nm}$ are defined as:

(D5) $$c_{nm}=\frac{1}{2N_{n-1,m}}[-\left(m+n\right)\left(m+n+1\right)N_{n,m+1}\left(K_{x,n,m+1}+iK_{y,n,m+1}\right)+N_{n,m-1}\left(K_{x,n,m-1}-iK_{y,n,m-1}\right)+2N_{n,m}\left(n+m\right)K_{z,n,m}]\text{.}$$

From this expansion, one can find the coefficients $a_{nm}$ defining the harmonic scalar function ψ as given in Eq. 19.
